# COVID-19 Neuromuscular Involvement in Post-Acute Rehabilitation

**DOI:** 10.3390/brainsci11121611

**Published:** 2021-12-06

**Authors:** Sergio Bagnato, Manfredi Ferraro, Cristina Boccagni, Gianluca Battaglia, Tiziana D’Agostino, Caterina Prestandrea, Marina Angela Bellavia, Francesca Rubino

**Affiliations:** 1 Unit of Neurophysiology, Rehabilitation Department, Giuseppe Giglio Foundation, 90015 Cefalù, Italy; manfredi.ferraro@hsrgiglio.it (M.F.); cristina.boccagni@hsrgiglio.it (C.B.); dagostinotiziana@gmail.com (T.D.); caterina.prestandrea@gmail.com (C.P.); marina.bellavia@hsrgiglio.it (M.A.B.); francesca.rubino@hsrgiglio.it (F.R.); 2 Unit of Cardiac and Pulmonary Rehabilitation, Rehabilitation Department, Giuseppe Giglio Foundation, 90015 Cefalù, Italy; gianluca.battaglia@hsrgiglio.it

**Keywords:** critical illness myopathy, critical illness polyneuropathy, Guillain-Barré syndrome, acute motor axonal neuropathy, demyelinating sensory-motor polyneuropathy, positioning nerve injury, peripheral nerve injury, neurological manifestations, electromyography, SARS-CoV-2

## Abstract

Background: Coronavirus disease 2019 (COVID-19) is associated with muscle and nerve injuries as a consequence of prolonged critical illness or the infection itself. In this study, we evaluated neuromuscular involvement in patients who underwent post-acute intensive rehabilitation after COVID-19. Methods: Clinical and neurophysiological evaluations, including nerve conduction studies and electromyography, were performed on 21 consecutive patients admitted for rehabilitation after COVID-19. Results: Clinical signs suggesting muscle or nerve involvement (weakness, reduced deep tendon reflexes, impaired sensitivity, abnormal gait) were found in 19 patients. Neurophysiological examinations confirmed neuromuscular involvement in 17 patients: a likely association of critical illness myopathy (CIM) and critical illness polyneuropathy (CIP) was found in 5 patients; CIM alone was found in 4 patients; axonal sensory-motor polyneuropathy was found in 4 patients (CIP in 2 patients, metabolic polyneuropathy in 2 patients); Guillain-Barré syndrome was found in 2 patients (classical demyelinating sensory-motor polyneuropathy and acute motor axonal neuropathy, respectively); peroneal nerve injury was found in 1 patient; and pre-existing L4 radiculopathy was found in 1 patient. Conclusions: Neuromuscular involvement is a very common finding among patients admitted for rehabilitation after COVID-19, and proper investigation should be conducted when muscle or nerve injury is suspected for adequate rehabilitative strategy planning.

## 1. Introduction

Coronavirus disease 2019 (COVID-19) has a wide range of neuromuscular involvement, from neuropathies to different degrees of muscle injury [1]. The polyneuropathies reported most commonly after COVID-19 are Guillain-Barré syndrome (GBS) and critical illness polyneuropathy (CIP). COVID-19-associated GBS has been estimated to be 5- to 13.5-fold more prevalent than GBS in the non–COVID-19 population [2,3]. GBS is considered to be the prototype for postinfectious neuropathy, reflecting the ability of some infectious agents to trigger an immune-mediated response against the peripheral nerves. Its clinical presentation is similar in patients with and without COVID-19, and the classical form of GBS, characterized by symmetrical weakness of the limbs, sensory symptoms, and reduced or absent tendon reflexes, is the manifestation reported most frequently after COVID-19 [4]. CIP is an axonal sensory-motor polyneuropathy that occurs in critically ill patients and it is a major cause of intensive care unit-acquired weakness [5]. In patients with severe COVID-19, CIP may be related to the rapid overproduction of cytokines and immune cell hyperactivation, together termed “cytokine release syndrome” or the “cytokine storm”, which is associated with various neurological manifestations of COVID-19 [6]. COVID-19 can also be related to muscle injuries, specifically critical illness myopathy (CIM), characterized by altered muscle excitability, muscle atrophy, and preferential myosin loss [5]. The pathophysiology of CIM is complex and partially overlaps that of CIP, with which CIM is often associated [5,7].

Neuromuscular involvement in patients with COVID-19 has been reported mainly when patients in intensive and semi-intensive care units develop weakness [8,9,10]; few cases have been described in the setting of post-acute rehabilitation [11,12], possibly because mild or moderate weakness can be misdiagnosed as an effect of long bed rest. However, the presence of neuropathies or myopathies, in addition to the common respiratory sequalae of COVID-19, necessitates longer rehabilitative hospitalization and specific rehabilitative programs.

Muscle and nerve damage may be easily revealed by standard neurophysiological evaluations, including nerve conduction studies and concentric needle electromyography (EMG). The aim of this study was to evaluate the presence of neuromuscular involvement in patients admitted for rehabilitation after COVID-19. The ability to identify such involvement has important practical implications for decision-making about the most appropriate rehabilitative strategies to apply for the promotion of recovery.

## 2. Materials and Methods

### 2.1. Patients

We recruited 21 consecutive patients (6 females, mean age 62.9 ± 13.4 years) admitted to the Giuseppe Giglio Foundation (Cefalù, Italy) for rehabilitation after COVID-19. Inclusion criteria were: (1) age ≥ 18 years; (2) COVID-19 diagnosis based on nasopharyngeal swab testing for severe acute respiratory syndrome coronavirus 2 (SARS-CoV-2) RNA, and chest computed tomography showing interstitial pneumonia; (3) hospitalization after COVID-19 diagnosis; and (4) the ability to provide informed consent to study participation. Exclusion criteria were: (1) previous evidence of myopathies or polyneuropathies; and (2) unstable clinical condition, such as hemodynamic instability or the requirement for invasive mechanical ventilation. All patients were admitted to rehabilitation after clearing SARS-CoV-2 infection, as demonstrated by negative results of nasopharyngeal swab testing for SARS-CoV-2 RNA. During hospitalization for rehabilitation, all patients underwent a personalized physiotherapy program for 180 min a day, 5 days a week.

### 2.2. Clinical and Neurophysiological Examinations

Clinical and neurophysiological evaluations of each patient were performed on the same day within 1 week after rehabilitation admission. The clinical evaluation encompassed a standard neurological examination, and the administration of the functional independence measure (FIM) and 6-min walk test (6MWT). The FIM is an 18-item, seven-level ordinal scale used to assess a patient’s degree of disability and functional change in response to rehabilitation; it was developed for application to a variety of patients, and is not diagnosis specific [13,14]. The 6MWT is a safe and well-standardized test used primarily to measure outcomes in people with moderate to severe heart or lung disease [15]. Patients’ functional outcomes were revaluated with the FIM and 6MWT at the time of discharge from rehabilitation.

The neurophysiological evaluation comprised nerve conduction studies and concentric needle EMG. All neurophysiological studies were performed with a Micromed (Mogliano Veneto, Italy) System Plus Evolution electromyograph. The ulnar, deep peroneal, and sural nerves were studied bilaterally in typical evaluations; more detailed examinations of some patients were performed. Surface electrodes were used for stimulation and recording. The electrical stimulation intensity (0.1-ms stimulus duration) was increased gradually to reach the maximal response amplitude, then increased an additional 20% to achieve supramaximal stimulation. The motor nerve study measurements performed were compound muscle action potential (CMAP) peak-to-peak amplitudes and latencies in the hands and feet in response to distal nerve stimulation, and the motor conduction velocity in the forearm or leg. CMAPs were recorded from the abductor digiti minimi and extensor digitorum brevis muscles after stimulation of the ulnar and deep peroneal nerves at the wrist and ankle at distances of 6 cm and 8 cm, respectively, from the recording electrodes placed at the centers of the muscle bellies. The sensory nerve conduction measurements taken were the sensory nerve action potential amplitude in response to antidromic nerve stimulation, and the sensory conduction velocity after stimulation of the ulnar nerve at the elbow and wrist, and the sural nerve in the distal third of the calf. Qualitative concentric-needle EMG was performed on the proximal and distal muscles of the upper and lower limbs, and spontaneous activity (such as fibrillation potentials and positive sharp waves) and voluntarily activated motor unit action potentials (MUAPs) were evaluated. Neurophysiological diagnoses of polyneuropathy and myopathy were made according to common standard criteria [16].

Clinical and neurophysiological data are reported descriptively for each patient.

## 3. Results

### 3.1. Patient Characteristics

The patients’ clinical details at the time of rehabilitation admission are reported in Table 1. Most patients had required non-invasive or invasive ventilation during the acute phase of COVID-19; the mean duration of hospitalization before admission to rehabilitation was 59.5 ± 25.2 days. Nineteen of the twenty-one patients (90.5%) had moderate or severe dyspnea at the time of neurophysiological evaluation, and most of them required oxygen therapy and/or non-invasive ventilation (Table 2). Clinical signs suggesting neuromuscular involvement (weakness, reduced deep tendon reflexes, impaired sensitivity, and/or abnormal gait) were observed in 19 of the 21 patients (90.5%); neurological examinations were normal for patients 10 and 17 (Table 2). In detail, weakness in the upper or lower limbs was present in 18 patients (87.5%), reduced deep tendon reflexes in the upper or lower limbs were present in 13 patients (61.9%), impaired sensation (to pain or vibration, or paresthesia/dysesthesia) was present in 9 patients (42.9%), and abnormal gait was present in 18 patients (85.7%). The mean duration of rehabilitative hospitalization was 57.1 ± 52.9 days, and all patients showed functional improvement during this hospitalization, as reflected by higher FIM scores and 6MWT distances at the time of discharge (Table 3).

### 3.2. Neurophysiological Findings

The main neurophysiological findings are reported according to main diagnoses in Table 4. Findings were pathological in 17 of the 21 patients; they were normal in patients 7, 10, 13, and 17.

#### 3.2.1. CIM Associated with CIP

Concurrent myopathic and neuropathic signs were detected in patients 2, 4, 5, 8, and 14. They were the most common pathological findings in our sample, suggesting an association of CIM and CIP. In detail, typical neurophysiological findings indicating myopathy (small MUAPS with early recruitment in the proximal muscles) were associated with signs of polyneuropathy (pathological nerve conduction, and large MUAPs with reduced recruitment in the distal muscles). Myopathic involvement was predominant over neuropathic involvement in patients 2 and 4, and prevalent involvement of both common peroneal nerves was demonstrated in patient 5.

#### 3.2.2. CIM

Myopathic findings alone, likely caused by CIM, were obtained for patients 3, 6, 12, and 21. Typical neurophysiological findings were small MUAPS with early recruitment in the lower or lower and upper limbs, associated with normal sensory nerve conduction findings. Patients 3 and 12 also showed reduced CMAP amplitudes in the lower limbs. In patient 4, who was unable to properly cooperate during EMG, the myopathic involvement was further supported performing direct muscle stimulation, which showed muscle inexcitability in the right quadriceps, and reduced CMAP amplitude in the right tibialis anterior. At the time of the neurophysiological evaluation, serum creatine kinase levels were normal in all patients.

#### 3.2.3. Axonal Sensory-Motor Polyneuropathy

Axonal sensory-motor polyneuropathy was diagnosed in patients 9, 15, 18, and 20. Based on anamnestic data, patients 9 and 18 likely had metabolic (diabetic) polyneuropathy, and patients 15 and 20 likely had CIP.

#### 3.2.4. GBS

GBS was diagnosed prior to rehabilitation admission in patients 11 and 16. Patient 11 developed a flu-like syndrome and, 2 weeks later, right eyelid ptosis, dysphagia, four-limb paresthesia, muscle pain, and abnormal gait. A nasopharyngeal swab test for SARS-CoV-2 administered upon admission to the emergency room was positive, and the patient was hospitalized first in the infectious disease unit, and then in neurology. The diagnosis of GBS was made based on cerebrospinal fluid analysis, nerve conduction studies, EMG, and brain and spinal-cord magnetic resonance imaging data. During hospitalization in the neurology unit, the patient was treated with intravenous immunoglobulin. After admission to rehabilitation, nerve conduction studies and EMG were repeated as part of this research protocol and demyelinating sensory-motor polyneuropathy was confirmed. The patient had mild clinical involvement, and a good outcome at discharge.

Patient 16 was first diagnosed with interstitial pneumonia associated with SARS-CoV-2 positivity (determined by nasopharyngeal swab testing), and she received standard care at home. About 9 days later, the patient developed severe four-limb weakness, and required hospitalization in the infectious disease unit; nasopharyngeal swab testing still indicated SARS-CoV-2 positivity. The diagnosis of the acute motor axonal neuropathy variant of GBS was made based on cerebrospinal fluid analysis, nerve conduction studies, EMG, and brain and spinal-cord magnetic resonance imaging data. Cerebrospinal fluid testing for SARS-CoV-2 RNA was negative. The patient was judged ineligible for intravenous immunoglobulin treatment due to the concomitant COVID-19. After admission to rehabilitation, nerve conduction studies and EMG were repeated, and confirmed the diagnosis of acute motor axonal neuropathy. The patient was tetraplegic at admission, required a long stay in rehabilitation, and still exhibited severe weakness in the lower limbs, and only limited improvement in the upper limbs at the time of discharge.

#### 3.2.5. Positioning Nerve Injury and Other

Lesions of both common peroneal nerves, with left-side predominance, were found in patient 19. They were likely caused by the patient’s positioning during the acute phase of COVID-19, with common peroneal nerve compression at the fibular head. Patients 5 and 9 also showed prevalent involvement of the common peroneal nerve in the contexts of concomitant CIM and CIP (patient 5), and axonal sensory-motor polyneuropathy (patient 9). Neurophysiological examination demonstrated pre-existing bilateral L4 radiculopathy in patient 1.

## 4. Discussion

In this study, neurophysiological examinations yielded abnormal findings in 81% of patients admitted for rehabilitation after COVID-19. These findings reflect a large spectrum of clinical and neurophysiological conditions, ranging from isolated mononeuropathies with limited functional impairment to polyneuropathies and myopathies with severe weakness and relevant functional impairment.

The most common form of neuromuscular involvement was intensive care unit–acquired weakness, including CIM and CIP. CIM associated with CIP was found in five patients; CIM alone (four patients) and CIP alone (two patients) were less common. Several factors may predispose patients with COVID-19 to the development of CIM and/or CIP; they include systemic inflammatory response with massive cytokine release, long duration of mechanical ventilation, and the use of neuromuscular blocking agents [5,17]. In the presence of myopathic involvement, diagnoses other than CIM, including sepsis-induced myopathy and steroid-denervation myopathy, should be considered [18]. These myopathies cannot be distinguished from CIM based on EMG data alone; further studies with muscle biopsy are needed for the detailed characterization of these patients’ myopathic involvement. Among the patients with axonal sensory-motor polyneuropathy, the most likely diagnoses were CIP and diabetic polyneuropathy in two cases each. However, these two conditions cannot be distinguished based on neurophysiological data alone; thus, we cannot exclude the possibility that all of these patients had CIP.

Our sample included two patients with different forms of GBS (demyelinating sensory-motor polyneuropathy, and acute motor axonal neuropathy) that were related temporally to SARS-CoV-2 infection. Current knowledge is insufficient to determine whether SARS-CoV-2 infection caused GBS in these patients, or whether these cases represent simple temporal concomitance. As in non-COVID-19 patients, GBS clinical course was more severe in the patient with acute motor axonal neuropathy than in the patient with demyelinating sensory-motor polyneuropathy.

Injury of the common peroneal nerve at the level of the fibular head was found in three patients (isolated in one patient, and in the context of polyneuropathy in two patients). Compression of the common peroneal nerve at the fibular head is the most common lower limb mononeuropathy, and its occurrence during hospitalization can be favored by weight loss, prolonged immobility, and lateral decubitus positioning [19].

Notably, clinical signs of neuromuscular involvement were found in all patients with abnormal neurophysiological findings. Two other patients showed mild weakness in the lower limbs with abnormal gait, but had normal nerve conduction study and EMG findings; these patients’ weakness was probably caused by prolonged bed stays during the acute phase of COVID-19.

The neurophysiological evaluations conducted in this study enabled accurate characterization of the type and severity of neuromuscular involvement after COVID-19, hence, allowing the adoption of proper rehabilitative strategies and refinement of prognoses. Moreover, all patients showed functional improvement (reflected by higher FIM scores and better 6MWT performance) at the time of discharge from rehabilitation relative to their conditions at the time of admission. These results support the relevance of the role of rehabilitation after severe COVID-19.

This study has some limitations. First, the sample was small, and the patients were recruited from a single center; thus, our findings cannot be generalized to all patients requiring rehabilitation after COVID-19 in different clinical contexts. Moreover, we did not perform muscle or nerve biopsies to better characterize myopathic and neuropathic involvement. Finally, most patients had dyspnea upon entry to the study, but we did not evaluate the phrenic nerve or the diaphragm. This could be an interesting topic to address in future studies in patients with neuromuscular involvement after COVID-19

## 5. Conclusions

This study showed that neuromuscular involvement is very common among patients who require rehabilitation after COVID-19. As nerve conduction studies and EMG are simple, inexpensive, and generally well-tolerated techniques, neurophysiological examination should be conducted at the beginning of rehabilitative treatment in all patients with clinical suspicion of neuromuscular involvement. Based on the findings of such examination, specific rehabilitative treatments to promote the best recovery possible after COVID-19 can be implemented.

## Figures and Tables

**Table 1:** Patients’ demographic and clinical details.

Pt	Sex	Age	Hospital Ward(s) before Admission in Rehabilitation	Mechanical Ventilation before Admission in Rehabilitation	Time between First Access to Emergency Room and Admission in Rehabilitation (Days)	Pre-Existing Risk Factors for Neuromuscular Involvement
1	M	84	Infectious disease unit, pulmonology	Yes	46	Diabetes
2	M	48	Pulmonology, ICU, medicine	Yes	134	None
3	F	62	Infectious disease unit, ICU, pulmonology	Yes	61	None
4	M	72	Infectious disease unit, ICU, pulmonology	Yes	107	None
5	M	64	Medicine, ICU	Yes	47	None
6	M	46	ICU	Yes	33	None
7	M	64	ICU	Yes	72	None
8	M	65	ICU	Yes	57	None
9	F	39	Medicine, ICU	Yes	62	Diabetes
10	F	72	Medicine	Yes	37	None
11	M	62	Infectious disease unit, neurology	No	56	None
12	F	79	Pulmonology	Yes	60	None
13	M	73	Infectious disease unit	No	41	None
14	M	61	Medicine, ICU	Yes	50	None
15	M	74	ICU, infectious disease unit	Yes	50	None
16	F	49	Infectious disease unit	No	27	None
17	M	76	Medicine	No	69	Diabetes
18	M	70	Pulmonology	Yes	41	Diabetes
19	M	58	Pulmonology	Yes	65	None
20	M	71	Medicine	No	43	None
21	F	33	ICU, pulmonology	Yes	69	None

Pt, patient.

**Table 2:** Respiratory and neurological clinical data at the time of neurophysiological examination.

Pt	Dyspnea	Oxygen Therapy	Mechanical Ventilation	Weakness		Reduced Deep Tendon Reflexes		Impaired Sensation			Abnormal Gait
				UL	LL	UL	LL	Pain (Blunt Tip Needle)	Vibration	Paresthesia/Dysesthesia	
1	3	Yes	No	2	1	0	2	0	0	0	2
2	3	Yes	Yes	1	2	1	1	0	0	0	3
3	3	Yes	No	1	2	0	1	0	0	0	2
4	3	No	No	1	2	1	1	0	0	0	3
5	3	Yes	No	0	2	0	3	1	0	0	2
6	3	Yes	No	0	1	0	0	0	0	0	0
7	3	Yes	No	0	1	0	0	0	0	0	1
8	3	No	No	1	1	0	0	0	0	0	1
9	2	No	No	1	3	0	2	1	0	0	1
10	2	No	Yes	0	0	0	0	0	0	0	0
11	0	No	No	0	1	1	1	1	1	1	1
12	3	Yes	No	1	2	0	1	0	1	0	3
13	2	No	No	0	1	0	0	0	0	0	1
14	3	Yes	No	2	1	2	2	1	1	0	2
15	3	Yes	No	0	1	0	1	0	1	0	3
16	0	No	No	3	3	3	3	0	0	0	3
17	3	Yes	No	0	0	0	0	0	0	0	0
18	3	Yes	No	0	1	2	2	1	1	0	1
19	3	Yes	Yes	0	2	0	0	1	0	2	1
20	1	No	No	0	0	0	1	0	1	0	1
21	2	No	No	1	1	0	0	0	0	0	1

Pt, patient; UL, upper limbs LL, lower limbs. 0 absent, 1 mild, 2 moderate, 3 severe. Score 3 arbitrarily corresponds to dyspnea at rest, paralysis, no reflexes, anesthesia to pain and vibration, and to the worst possible paresthesia/dysesthesia.

**Table 3:** Functional evaluation results at the times of admission and discharge.

Pt	Hospitalization in Rehabilitation (Days)	Functional Independence Measure Scores		6-Min Walk Test ^1^	
		At Admission	At Discharge	At Admission	At Discharge
1	35	89	110	80 (19%)	270 (65%)
2	151	24	44	Not feasible	Not feasible
3	69	45	104	Not feasible	Not feasible
4	62	42	56	Not feasible	Not feasible
5	36	89	106	Not feasible	330 (63%)
6	36	96	125	Not feasible	540 (85%)
7	55	80	105	Not feasible	540 (100%)
8	41	74	118	Not feasible	300 (59%)
9	31	117	126	280 (42%)	450 (67%)
10	37	118	126	250 (56%)	380 (85%)
11	20	120	126	420 (77%)	570 (105%)
12	64	90	110	Not feasible	210 (49%)
13	45	84	126	Not feasible	480 (89%)
14	52	72	120	Not feasible	450 (83%)
15	17	78	100	Not feasible	Not available
16	252	45	55	Not feasible	Not feasible
17	41	85	116	Not feasible	180 (41%)
18	47	90	122	Not feasible	390 (78%)
19	65	24	126	Not feasible	290 (54%)
20	28	106	116	120 (29%)	210 (50%)
21	15	122	126	190 (27%)	360 (51%)

Pt, patient. ^1^ Results are reported as meters (percentage of expected normal value, adjusted for age and sex).

**Table 4:** Main neurophysiological findings.

Pt	Reduced CMAP Amplitude		Reduced MCV		Reduced SNAP Amplitude		Reduced SCV		Denervation		Myopathic MUAPs		Neurogenic MUAPs		Neurophysiological Report
	UL	LL	UL	LL	UL	LL	UL	LL	UL	LL	UL	LL	UL	LL	
1	0	0	0	0	0	0	0	0	0	+	0	+	0	+	Chronic bilateral L4 radiculopathy
2	+	+	0	+	0	0	0	0	0	+	+	+	0	+	Critical illness myopathy and polyneuropathy with prevalent myopathic involvement
3	0	+	0	0	0	0	0	0	0	0	+	+	0	0	Critical illness myopathy
4	0	+	0	+	+	+	+	+	0	0	0	+	+	0	Critical illness myopathy and polyneuropathy with prevalent myopathic involvement
5	0	+	0	0	0	+	0	0	0	+	+	0	+	+	Critical illness myopathy and polyneuropathy with prevalent involvement of both common peroneal nerves
6	0	0	0	0	0	0	0	0	0	0	0	+	0	0	Mild critical illness myopathy
7	0	0	0	0	0	0	0	0	0	0	0	0	0	0	Normal examination
8	0	+	+	0	0	0	0	+	0	0	0	+	0	0	Mild critical illness myopathy and polyneuropathy
9	0	+	0	0	0	+	0	0	0	+	0	0	0	+	Mild axonal sensory-motor polyneuropathy with severe lesion of the right common peroneal nerve
10	0	0	0	0	0	0	0	0	0	0	0	0	0	0	Normal examination
11	0	+	+	+	+	+	+	+	0	0	0	0	+	+	Demyelinating sensory-motor polyneuropathy
12	0	+	0	0	0	0	0	0	0	0	+	+	0	0	Critical illness myopathy
13	0	0	0	0	0	0	0	0	0	0	0	0	0	0	Normal examination
14	+	+	+	0	+	0	+	0	+	0	+	+	+	+	Critical illness myopathy and polyneuropathy
15	0	+	+	0	0	+	+	+	NA	0	NA	0	NA	+	Mild critical illness polyneuropathy
16	+	+	+	+	0	0	0	0	+	+	0	No MUAPs	+	No MUAPs	Acute motor axonal polyneuropathy
17	0	0	0	0	0	0	0	0	0	0	0	0	0	0	Normal examination
18	0	+	+	+	+	+	+	+	0	0	0	0	+	+	Axonal sensory-motor polyneuropathy
19	0	+	0	0	0	+	0	0	0	+	0	0	0	+	Lesion of both common peroneal nerves, prevalent in the left side
20	0	+	0	0	0	+	0	0	0	0	0	0	0	+	Critical illness polyneuropathy
21	0	0	0	0	0	0	0	0	0	0	+	+	0	0	Critical illness myopathy

Pt, patient; CMAP, compound muscle action potential; MCV, motor conduction velocity; SNAP, sensory nerve action potential; SCV, sensory conduction velocity; MUAP, motor unit action potential; UL, upper limbs; LL, lower limbs; NA, not available. + present, 0 absent.

## Data Availability

The datasets generated during the current study are available from the corresponding author on reasonable request.
